# Dyslipidemia patterns and associated factors in Wilson’s disease patients: a clinical analysis

**DOI:** 10.3389/fneur.2024.1411236

**Published:** 2024-11-20

**Authors:** Rong Wu, Xingguang Luo, Xiao-Ping Wang

**Affiliations:** ^1^Department of Neurology, Tongren Hospital, School of Medicine, Shanghai Jiao Tong University, Shanghai, China; ^2^Department of Psychiatry, School of Medicine, Yale University, New Haven, CT, United States; ^3^Renji Hospital, Shanghai Jiao Tong University Medical School, Shanghai, China

**Keywords:** Wilson’s disease, dyslipidemia, influencing factors, regression analysis, lipidemia metabolism

## Abstract

**Objective:**

This study aims to analyze the lipid metabolism patterns and identify risk factors for dyslipidemia in Wilson’s Disease (WD), offering novel insights into diagnosis and treatment strategies for unexplained dyslipidemia.

**Methods:**

Data from Wilson’s disease patients hospitalized at the First People’s Hospital of Shanghai from December 2008 to February 2015 were collected. Patients were categorized into normal lipid (46 cases) and dyslipidemia (42 cases) groups based on lipid levels. Group analyzes were conducted using t-tests, chi-square analysis, and rank sum tests. Spearman correlation, multiple linear regression, or Logistic regression were employed to identify relevant influencing factors.

**Results:**

1. The incidence of abnormal blood lipids in a series of Wilson’s disease patients was 47.73% (25.12 ± 1.29 years old), and the incidence of control healthy group was 27.40%, with proportions of hypercholesterolemia, hypertriglyceridemia, and low-density lipoprotein cholesterol being 14.77, 30.68, and 29.63%, respectively; 2. Significant differences were observed between the dyslipidemia and normal WD groups in AST/ALT ratio, liver parenchymal echo, liver surface, spleen area, and ultrasound total score.3. Low-and high-density lipoprotein cholesterols (LDL-c and HDL-c) showed no significant correlation with these indicators. Triglyceride (TG) exhibited moderately negative correlation with AST/ALT, liver parenchymal echo, spleen area, and ultrasound score. Total cholesterol (TC) displayed low negative correlation with these factors.

**Conclusion:**

1. Dyslipidemia incidence in Wilson’s disease patients may exceed that of the normal population, especially in adolescents with unexplained abnormal lipid metabolism; 2. Patients with mild to moderate liver damage are predisposed to elevated triglycerides and total cholesterol, reflecting liver damage impact on lipid metabolism; 3. Glucose metabolism is not implicated in WD-related dyslipidemia; 4. No significant correlation was found between abnormal lipid metabolism and blood concentration of trace elements in WD patients.

## 1 Introduction

Hepatolenticular Degeneration (HLD), also known as Wilson’s Disease (WD), is an autosomal recessive genetic disorder characterized by impaired copper metabolism, leading to lesions in the liver, brain, kidneys, cornea, and other organs. It was initially described by Wilson in 1912. While the incidence rate in most European and American countries ranges from 15 to 30 per million, epidemiological data in China suggest a higher incidence (1). The pathogenesis of WD primarily involves mutations in the ATP7B gene located on chromosome 13. This gene encodes a copper-dependent P-type ATPase responsible for transporting copper to the Golgi apparatus, synthesizing plasma ceruloplasmin, and facilitating copper excretion. Mutations in ATP7B disrupt copper binding to ceruloplasmin, leading to copper accumulation in the body, particularly in the central nervous system and liver.

WD typically manifests in childhood or early adulthood, with liver dysfunction usually appearing around 11 years of age and neurological symptoms around 19 years. Left untreated, WD progresses to involve both liver and neurological systems. Rare manifestations may include joint disorders, fever, hemolytic anemia, and behavioral abnormalities. Diagnosis relies on a combination of clinical symptoms, laboratory tests, genetic analysis, and imaging studies. The 2012 EASL guidelines recommend considering WD in patients with unexplained liver dysfunction or newly diagnosed motor disorders. Diagnostic evaluation includes medical history, physical examination, liver function tests, serum copper and ceruloplasmin levels, 24-h urine copper excretion, liver biopsy for copper quantification, slit lamp examination for K-F rings, and family history screening.

Abnormal blood lipid metabolism can have secondary or primary causes (2). Secondary dyslipidemia is often associated with systemic diseases such as diabetes, nephrotic syndrome, and hypothyroidism, while primary dyslipidemia may result from congenital genetic defects or other unknown factors. Clinical observations indicate that WD patients frequently exhibit dyslipidemia, and some adolescents with unexplained dyslipidemia are ultimately diagnosed with WD after thorough evaluation. Therefore, we conducted a retrospective analysis of patient medical records to investigate the prevalence of abnormal blood lipids and identify potential influencing factors, aiming to offer insights into the diagnosis and management of unexplained dyslipidemia in clinical practice.

## 2 Materials and methods

### 2.1 Research participants

#### 2.1.1 Dyslipidemia group

This study was approved by the local ethical committee, and all participants signed an informed consent form. Patients diagnosed with hepatolenticular degeneration and admitted to the First People’s Hospital of Shanghai between December 2008 and February 2015 were included. Inclusion criteria: (1) Diagnosis meeting the European Association for the Study of the Liver (EASL) criteria for hepatolenticular degeneration (2011) (3); (2) Underwent at least one blood lipid test during hospitalization; (3) Blood lipid test results indicating at least one of the following: LDL-c > 4.41 mmoL/L, HDL-c < 1.03 mmoL/L, TG > 1.70 mmoL/L, or TC > 5.20 mmoL/L. Exclusion criteria: (1) Dyslipidemia due to other causes; (2) Significant data loss; (3) Concurrent diseases affecting elemental metabolism such as copper and zinc; (4) Liver, brain, kidney, eye, or heart diseases unrelated to hepatolenticular degeneration; (5). Have taken or are currently taking medications that affect blood lipid levels.

#### 2.1.2 Normal lipid group

Patients diagnosed with hepatolenticular degeneration and admitted to the First People’s Hospital of Shanghai between December 2008 and February 2015 were included. Inclusion criteria: (1) Diagnosis meeting the EASL criteria for hepatolenticular degeneration (2011) (3); (2) Underwent at least one blood lipid test during hospitalization; (3) Blood lipid test results indicating LDL-c levels of 1.30–4.41 mmoL/L, HDL-c levels of 1.03–1.55 mmoL/L, TG levels below 1.70 mmoL/L, and TC levels below 5.20 mmoL/L. Exclusion criteria: (1) Dyslipidemia due to other causes; (2) Significant data loss; (3) Concurrent diseases affecting elemental metabolism such as copper and zinc; (4) Liver, brain, kidney, eye, or heart diseases unrelated to hepatolenticular degeneration (5). Have taken or are currently taking medications that affect blood lipid levels. Diagnosed patients were all prescribed a low-copper diet and were treated with copper-chelating drugs orally or intravenously, with monitoring of urinary copper levels. All patients were managed using a standardized clinical pathway.

### 2.2 Research methods

Neurologists collected relevant data from enrolled patients, including disease duration, age, gender, LDL-c, HDL-c, TC, TG, *γ*-Glutamyltransferase (γ-GT), alanine aminotransferase (ALT), alkaline phosphatase (ALP), lactate dehydrogenase (LDH), aspartate aminotransferase (AST), AST/ALT ratio, blood glucose, blood trace elements, liver Nazer score (4), ultrasound score (5), etc.

#### 2.2.1 General data collection

Data on disease duration, age, gender, and medical history were collected by the same neurologist specializing in neurology for both the dyslipidemia group and the control group.

#### 2.2.2 Clinical data

##### 2.2.2.1 Blood lipids

Blood lipid levels were measured before intravenous copper therapy using the Johnson & Johnson VITROS 4600 biochemical immunoassay analyzer. Fasting venous blood samples (2-3 mL) were collected in the morning and analyzed on the same day. Patients were categorized based on lipid levels: dyslipidemia if any indicator exceeded the normal range, and normal lipid group if all indicators fell within normal limits.

##### 2.2.2.2 Liver ultrasound scoring

Liver ultrasound examinations were conducted using the French Aixplorer ultrasound diagnostic instrument. Various parameters were assessed, and semi-quantitative scoring was performed based on predefined criteria (Table 1) (5). Scores ranged from 6 to 18, with higher scores indicating more severe liver damage.

**Table 1:** Integral division criteria for liver ultrasound parameters.

Item	1 point	2 points	3 points
Liver parenchymal echo	Fine and evenly distributed intrahepatic light spots	Echo intensity slightly increased, rough, uneven	The echo is significantly enhanced, with patchy, reticular, linear, or nodular strong echogenic light masses visible
Liver surface	Normal	Irregular	Serrated, wavy, or nodular
Liver margin	Normal	The tip becomes blunt, but the shape of the left lobe of the liver is normal	Extremely dull, the left lobe of the liver loses its normal shape
hepatic vein	Normal	Vague	Narrow, uneven in thickness or curved into a snake shape
Spleen area	Below 22 cm	22 cm-28 cm	Above 28 cm
gallbladder wall	Normal	Roughness	Obvious thickening or bilateral signs

##### 2.2.2.3 Liver enzymes

Liver enzyme indicators routinely assessed upon patient admission were included as research factors, comprising alanine aminotransferase (ALT), aspartate aminotransferase (AST), *γ*-Glutamyltransferase (γ-GT), alkaline phosphatase (ALP), lactate dehydrogenase (LDH), and AST/ALT ratio. Normal reference ranges provided by the hospital’s biochemical routine test kit were as follows: ALT: 5.0–40.0 U/L, AST: 8.0–40.0 U/L, *γ*-GT: 7.0–50.0 U/L, ALP: 35.0–129.0 U/L, LDH: 109.0–245.0 U/L, AST/ALT: 0.8–1.5.

##### 2.2.2.4 Blood glucose

Blood glucose levels were considered as a research factor due to the interplay between glucose and lipid metabolism. Many diseases affecting glucose metabolism can induce lipid metabolism abnormalities. For instance, insulin resistance and/or insulin secretion deficiency in diabetes patients often lead to dyslipidemia. Therefore, blood sugar levels were included in this study. The normal reference range for blood sugar was 3.90–6.10 mmol/L.

##### 2.2.2.5 Nazer liver function score

The Nazer liver function score comprises aspartate aminotransferase (AST), total bilirubin (TBIL), and international normalized ratio (INR), each categorized into five levels (0–4 points) for scoring. The sum of the scores from these three indicators yields the patient’s liver function score. Higher scores indicate more severe liver function impairment. A total score of 7 or higher often indicates severe liver function damage, warranting consideration for liver transplantation. Please refer to Table 2 for specific scoring criteria.

**Table 2:** Modified nazer liver function score.

Item	0	1	2	3	4
Total bilirubin (umol/L)	<100	100–150	151–200	201–300	>300
Aspartate aminotransferase (U/L)	<100	100–150	151–200	201–300	>300
International normalized ratio	<1.3	1.3–1.6	1.6–1.9	1.9–2.4	>2.4

##### 2.2.2.6 Blood trace elements

Despite being characterized by copper overload, patients with hepatolenticular degeneration often exhibit decreased serum copper levels (3), and may also present abnormal serum levels of various trace elements. Animal studies have demonstrated the influence of blood trace element levels on lipid metabolism (6). Therefore, this study included the assessment of blood trace element levels, primarily encompassing calcium, cadmium, magnesium, lead, iron, copper, zinc, and the copper/zinc ratio. Normal reference ranges are as follows: calcium 1.55–2.1 mmol/L, cadmium 0-5ug/L, magnesium 1.12–2.06 mmol/L, lead 0-100ug/L, iron 7.52–11.82 mmol/L, copper 11.8–39.3 μMol/L, zinc 76.5–170 μMol/L.

### 2.3 Statistical analysis

Statistical analyzes were performed using SPSS 17.0 software. Quantitative data are presented as mean ± standard deviation ( $\bar{x}$ ± s). Group t-tests were employed for intergroup comparisons of normally distributed data, while non-parametric rank sum tests were used for non-normally distributed data. Comparison of categorical data utilized the chi-square test (Fisher’s exact probability method). Mann–Whitney rank sum tests were conducted for comparison of two independent sample-level data. Correlation analysis employed the Spearman rank correlation test, with correlation coefficients categorized as follows: r < 0.3 indicates low correlation, 0.3 ≤ r < 0.6 indicates moderate correlation, and r ≥ 0.6 indicates high correlation. Multiple linear stepwise regression analysis was utilized to identify potential influencing factors for LDL-c, HDL-c, TC, and TG data. A significance level of *α* = 0.05 was used for testing differences.

Categorical variables were assigned values as follows: gender: male = 1, female = 2.

## 3 Result

### 3.1 Comparison of general information

Following the inclusion and exclusion criteria outlined in section 1.1, a total of 124 individuals with hepatolenticular degeneration were included. Due to severe incomplete data, 36 individuals were excluded, leaving a final total of 88 individuals. Among them, 67 individuals were included in the dyslipidemia group. Due to severe incomplete data, 25 individuals were excluded, leaving a final total of 42 individuals in the dyslipidemia group, comprising 19 males and 23 females, aged 4–46 years, with an average age of 22.29 ± 11.28 years (95% CI [18.77, 25.80]), and a disease duration of 1–21 years, averaging 7.43 ± 5.62 years (95% CI [5.68, 9.18]). Additionally, 57 individuals were included in the normal lipid group. Due to severe incomplete data, 11 individuals were excluded, leaving a final total of 46 individuals in the normal lipid group, including 28 males and 18 females, aged 4–62 years, with an average age of 24.96 ± 9.78 years [95% CI (22.05, 27.86)], and a disease duration of 1–34 years, averaging 8.25 ± 7.33 years [95% CI (6.07, 10.43)]. There were no significant differences in gender distribution, age, or disease duration between the two groups (*p* > 0.05), indicating comparability. We performed a multivariate regression analysis, utilizing gender as the independent variable and blood lipid levels as the dependent variable. The findings revealed that all *p*-values exceeded 0.05, suggesting that in our study, a significant regression relationship between gender and blood lipids has not been established. Therefore, we cannot conclude that gender has an impact on blood lipid levels.

### 3.2 Analysis of blood lipid indicators

Upon analyzing blood lipid indicators in enrolled patients, it was found that 42 WD patients exhibited abnormal blood lipids, constituting 47.73% of all enrolled individuals. Within this group, the proportions of hypercholesterolemia, hypertriglyceridemia, and low-density lipoprotein cholesterol were 13.64, 29.55, and 20.37%, respectively. The dyslipidemia group showed average values of 2.32 ± 0.70 mmoL/L for LDL-c, 1.14 ± 0.42 mmoL/L for HDL-c, 1.77 ± 0.61 mmoL/L for TG, and 4.31 ± 1.06 mmoL/L for TC. In contrast, the group with normal blood lipids exhibited average values of 1.89 ± 0.44 mmoL/L for LDL-c, 1.36 ± 0.22 mmoL/L for HDL-c, 0.86 ± 0.24 mmoL/L for TG, and 3.63 ± 0.54 mmoL/L for TC. Significant differences were observed between the two groups in LDL-c (*p* < 0.05), HDL-c (*p* < 0.05), TG (*p* < 0.01), and TC (*p* < 0.01). (Refer to Table 3 for details).

**Table 3:** Comparison of four blood lipid levels between the dyslipidemia group and the normal blood lipid group.

Item	Dyslipidemia group	Normal blood lipid group	*t*- values	*p*- value	95% CI
LDL-c(mmol/l)	2.32 ± 0.70	1.89 ± 0.44	−2.521	0.015	(−0.77,-0.09)
HDL-c(mmol/l)	1.14 ± 0.42	1.36 ± 0.22	2.237	0.03	(0.02,0.41)
TG(mmol/l)	1.77 ± 0.61	0.86 ± 0.24	−9.030	<0.01	(−1.10,-0.71)
TC(mmol/l)	4.31 ± 1.06	3.63 ± 0.54	−3.742	<0.01	(−1.04,-0.32)
BMI (kg/m2)	21.28 ± 1.43	21.84 ± 1.83	1.603	0.113	(−0.14, 1.27)

### 3.3 Analysis of ultrasound results

Utilizing the liver ultrasound parameter integration criteria, non-parametric rank sum tests were conducted on six sub-items: liver parenchymal echo, liver surface, liver edge, liver vein, spleen area, and gallbladder wall, culminating in the total ultrasound score.

In the dyslipidemia group, the average scores were as follows: liver parenchymal echo (1.81 ± 0.53 points), liver surface (1.17 ± 0.45 points), liver edge (1.00 ± 0.00 points), liver vein (1.11 ± 0.32 points), spleen area (1.75 ± 0.94 points), gallbladder wall (1.19 ± 0.40 points), and total ultrasound score (8.03 ± 1.73 points).

Conversely, in the group with normal blood lipids, the average scores were: liver parenchymal echo (2.10 ± 0.40 points), liver surface (1.39 ± 0.56 points), liver edge (1.10 ± 0.30 points), liver vein (1.03 ± 0.18 points), spleen area (2.26 ± 0.90 points), gallbladder wall (1.39 ± 0.62 points), and total ultrasound score (9.26 ± 1.84 points).

Significant differences were observed in liver parenchymal echo (*p* < 0.05), liver surface (*p* < 0.05), spleen area (*p* < 0.05), and total ultrasound score (*p* < 0.01) between the two groups.

### 3.4 Analysis of liver enzyme results

In the dyslipidemia group, *γ*-Glutamyltransferase (*γ*-GT) averaged 41.20 ± 26.77 U/L, alanine aminotransferase (ALT) averaged 52.06 ± 67.99 U/L, alkaline phosphatase (ALP) averaged 140.20 ± 95.83 U/L, lactate dehydrogenase (LDH) averaged 180.37 ± 75.19 U/L, aspartate aminotransferase (AST) averaged 35.22 ± 25.21 U/L, and the average AST/ALT ratio was 0.93 ± 0.44.

In the normal blood lipid group, γ-Glutamyltransferase (γ-GT) averaged 43.74 ± 44.14 U/L, alanine aminotransferase (ALT) averaged 30.69 ± 44.06 U/L, alkaline phosphatase (ALP) averaged 127.17 ± 119.23 U/L, lactate dehydrogenase (LDH) averaged 160.46 ± 47.09 U/L, aspartate aminotransferase (AST) averaged 33.24 ± 38.53 U/L, and the average AST/ALT ratio was 1.41 ± 0.65.

A significant difference (*p* < 0.01) was observed in AST/ALT between the two groups. However, there were no significant differences in *γ*-GT, ALT, AST, ALP, and LDH indicators (*p* > 0.05; Refer to Table 4 for details).

**Table 4:** Comparison of liver enzyme indicators between the group with the dyslipidemia group and the normal blood lipid group.

Item	Dyslipidemia group	Normal blood lipid group	*t*- values	*p*- value	95% CI
*γ*-Glutamatyltransferase (U/L)	41.20 ± 26.77	43.74 ± 44.14	0.320	0.750	(−13.26,18.34)
Alanine aminotransferase (U/L)	52.06 ± 67.99	30.69 ± 44.06	−1.758	0.082	(−45.53,2.80)
Alkaline phosphatase (U/L)	140.20 ± 95.83	127.17 ± 119.23	−0.557	0.579	(−59.50,33.46)
Lactate dehydrogenase (U/L)	180.37 ± 75.19	160.46 ± 47.09	−1.497	0.138	(−46.35,6.53)
Aspartate aminotransferase (U/L)	35.22 ± 25.21	33.24 ± 38.53	−0.279	0.781	(−16.04,12.09)
AST/ALT	0.93 ± 0.44	1.41 ± 0.65	4.045	<0.01	(0.24,0.71)

### 3.5 Analysis of blood glucose results

The average blood glucose level in the dyslipidemia group was 4.53 ± 0.46 mmoL/L, whereas in the normal blood lipid group, it was 4.44 ± 0.85 mmoL/L. No significant difference was observed between the groups [*p* > 0.05, 95%CI (−0.39,0.20)], suggesting comparable blood glucose levels between the two groups.

### 3.6 Nazer score analysis

The average Nazer score in the dyslipidemia group was 0.02 ± 0.16, compared to 0.16 ± 0.53 in the normal lipidemia group. No significant difference was noted between the groups (*p* > 0.05), indicating a lack of distinction in Nazer scores between the dyslipidemia and normal groups.

### 3.7 Trace element analysis

In the dyslipidemia group, the average serum calcium content was 1.64 ± 0.12 mmol/L, cadmium content averaged 1.44 ± 1.29ug/L, magnesium content averaged 1.51 ± 0.18 mmol/L, lead content averaged 39.39 ± 21.13ug/L, iron content averaged 8.53 ± 1.03 mmol/L, copper content averaged 11.62 ± 4.25 μMol/L, zinc content averaged 89.37 ± 13.72 μMol/L, and the copper/zinc ratio averaged 0.10 ± 0.07.

In the normal blood lipid group, the average serum calcium content was 1.64 ± 0.12 mmol/L, cadmium content averaged 1.44 ± 1.29ug/L, magnesium content averaged 1.51 ± 0.18 mmol/L, lead content averaged 39.39 ± 21.13ug/L, iron content averaged 8.53 ± 1.03 mmol/L, copper content averaged 11.62 ± 4.25 μMol/L, zinc content averaged 89.37 ± 13.72 μMol/L, and the copper/zinc ratio averaged 0.10 ± 0.07.

There were no significant differences (*p* > 0.05) observed in the comparison of various trace elements and the copper/zinc ratio between the groups. Hence, no significant disparities were found in serum calcium, cadmium, magnesium, lead, iron, copper, zinc, and the copper/zinc ratio between the dyslipidemia and normal groups.

### 3.8 Correlation analysis

Upon comparison between the dyslipidemia group and the normal lipidemia group, significant disparities were observed in liver parenchymal echo, liver surface, spleen area, total ultrasound score, and AST/ALT indicators. Consequently, these five variables were identified as candidate risk factors, and the correlation analysis results are outlined as follows (see Table 5):

**Table 5:** Correlation analysis of four blood lipids and research factors in hepatolenticular degeneration.

Item		LDL-c	HDL-c	TG	TC
AST/ALT	*r_s_*	−0.208	0.026	−0.399^**^	−0.248^*^
	*p*- value	0.131	0.853	0.000	0.020
	95% CI	−0.46,0.07	−0.25,0.30	−0.57,-0.20	−0.44,-0.04
Liver parenchymal echo	*r_s_*	0.007	0.014	−0.320^**^	−0.247^*^
	*p*- value	0.962	0.925	0.008	0.044
	95% CI	−0.29,0.30	−0.28,0.31	−0.53,-0.08	−0.47,0.00
Liver surface	*r_s_*	0.044	0.130	−0.105	0.014
	*p*- value	0.770	0.384	0.398	0.907
	95% CI	−0.26,0.34	−0.17,0.41	−0.34,0.15	−0.23,0.26
Spleen area	*r_s_*	−0.163	0.103	−0.396^**^	−0.292^*^
	*p*- value	0.272	0.490	0.001	0.017
	95% CI	−0.44,0.14	−0.20,0.39	−0.59,-0.17	−0.50,-0.05
Ultrasound score	*r_s_*	−0.165	0.178	−0.352^**^	−0.260^*^
	*p*- value	0.269	0.231	0.003	0.034
	95% CI	−0.44,0.14	−0.12,0.45	−0.55,-0.12	−0.48,-0.14

^*^p < 0.05; ^**^p < 0.01 in the table.

#### 3.8.1 Low-density lipoprotein cholesterol

Correlation analysis of LDL-c indicators across all enrolled WD patients revealed no significant correlations with liver parenchymal echo score, liver surface score, spleen area score, total ultrasound score, or AST/ALT ratio.

#### 3.8.2 High-density lipoprotein cholesterol

Correlation analysis of HDL-c indicators among all enrolled WD patients showed no notable correlations with liver parenchymal echo score, liver surface score, spleen area score, total ultrasound score, or AST/ALT ratio.

#### 3.8.3 Serum triglycerides

Correlation analysis of TG indicators among all enrolled WD patients indicated moderate negative correlations with AST/ALT (*r* = −0.399, *p* < 0.01), liver parenchymal echo (*r* = −0.320, *p* < 0.01), spleen area (*r* = −0.396, *p* < 0.01), and ultrasound score (*r* = −0.352, *p* < 0.01), while no correlation was found with liver surface score.

#### 3.8.4 Total serum cholesterol

Correlation analysis of TC indicators among all enrolled WD patients revealed a low degree of negative correlation between TC and AST/ALT (*r* = −0.248, *p* < 0.05), liver parenchymal echo (*r* = −0.247, *p* < 0.05), spleen area (*r* = −0.292, *p* < 0.05), and ultrasound score (*r* = −0.260, *p* < 0.05), with no correlation observed with liver surface score.

### 3.9 Regression analysis of influencing factors

Five research factors, including liver parenchymal echo, liver surface, splenic area, total ultrasound score, and AST/ALT index, were utilized as independent variables, while blood lipids served as dependent variables for regression analysis of influencing factors.

#### 3.9.1 Low-density lipoprotein cholesterol

Multiple linear stepwise regression analysis was conducted with liver parenchymal echo score, liver surface score, spleen area score, total ultrasound score, and AST/ALT value as independent variables, and LDL-c as the dependent variable. The analysis revealed no independent variables selected into the equation.

#### 3.9.2 High-density lipoprotein cholesterol

Multiple linear stepwise regression analysis was performed with liver parenchymal echo score, liver surface score, spleen area score, total ultrasound score, and AST/ALT value as independent variables, and HDL-c as the dependent variable. Similar to LDL-c, no independent variables were selected into the equation.

#### 3.9.3 Serum triglycerides

Multiple linear stepwise regression analysis was conducted with liver parenchymal echo score, liver surface score, spleen area score, total ultrasound score, and AST/ALT value as independent variables, and TG values as the dependent variable. The results indicated that spleen area and AST/ALT ratio were selected as independent variables into the equation: YTG = 2.290–0.398X spleen area-0.314 XAST/ALT, with t-values of-2.690 and-2.399, respectively, both significant at *p* < 0.01 and *p* < 0.05. The coefficient of determination R^2^ was 0.172, suggesting that spleen area and AST/ALT ratio account for 17.2% of the variation in TG levels.

#### 3.9.4 Total serum cholesterol

Using TC values as the dependent variable, multiple linear stepwise regression analysis was performed with liver parenchymal echo score, liver surface score, spleen area score, total ultrasound score, and AST/ALT value as independent variables. The results revealed that spleen area was selected as the independent variable in the equation: YTC = 4.962–0.631 X spleen area, with a t-value of-2.771, significant at *p* < 0.01. The coefficient of determination *R*^2^ was 0.092, indicating that spleen area contributes to 9.2% of the variation in TC levels.

## 4 Discussion

### 4.1 Analysis of blood lipid levels in patients with hepatolenticular degeneration

This study included 88 patients with Wilson’s disease (WD), of whom 42 exhibited dyslipidemia, constituting 47.73% of the enrolled cohort, while the incidence of abnormal blood lipids of control healthy group was 27.40%. Among them, the prevalence of hypercholesterolemia, hypertriglyceridemia, and elevated low-density lipoprotein cholesterol (LDL-c) levels were notably higher compared to the general population (7). Hence, when encountering unexplained dyslipidemia, particularly in young patients, clinicians should consider hepatolenticular degeneration as a potential underlying cause during routine diagnostic evaluations.

The precise mechanism responsible for the development of dyslipidemia in WD patients remains incompletely understood. Early studies in Atp7b−/− mice suggest that copper is initially sequestered by metallothionein in the cytoplasm. As copper accumulates, it translocates to the nucleus, triggering transcriptome remodeling that disrupts the cell cycle and lipid metabolism (8). Additional research has demonstrated that ATP7B is expressed in intestinal epithelial cells and plays a crucial role in regulating copper vesicle storage, potentially influencing intestinal lipid metabolism (9). Moreover, excessive copper accumulation can lead to alterations in mitochondrial morphology and function, with signs of mitochondrial damage observed in both humans and animal models of WD, often preceding hepatic dysfunction (10–15). It is speculated that WD may impact lipid metabolism through perturbations in the tricarboxylic acid cycle (TCA) and related pathways (16), as changes in TCA cycle intermediates have been previously documented in WD patients. Based on a review of the literature, it is hypothesized that dyslipidemia in WD patients may be linked to copper overload and its effects on lipid metabolism-related pathways, as well as the modulation of intestinal lipid metabolism by ATP7B (17).

### 4.2 Analysis of factors related to blood lipid levels in patients with hepatolenticular degeneration

#### 4.2.1 Liver ultrasound

Significant disparities were observed in liver parenchymal echo, liver surface, spleen area, and total ultrasound score between the dyslipidemia group and the normal blood lipid group in this study. Notably, a moderate negative correlation was identified between serum triglycerides and liver parenchymal echo, spleen area, and total ultrasound score. Similarly, serum total cholesterol exhibited negative correlations with liver parenchymal echo, spleen area, and overall ultrasound score.

Interpreting ultrasound scores based on defined criteria, an echo greater than 1 point in liver parenchyma or a surface score exceeding 1 point suggests liver fibrosis or even cirrhosis. Liver cell damage precipitates degeneration, necrosis, and inflammation, initiating fibrosis characterized by reticular fiber collapse and subsequent collagenization. As fibrous tissue connects and remodels liver lobules, cirrhosis ensues. Increased liver cell necrosis correlates with reduced lipid synthesis, contributing to diminished blood lipid levels. Hepatic fibrosis is a intricate process entailing interactions among diverse populations of resident and non-resident hepatocytes, ultimately culminating in the deposition of extracellular matrix and subsequent organ failure (18). The liver holds a pivotal and distinctive position in lipid metabolism, functioning as a indispensable organ for the synthesis, catabolism, and metabolism of lipids (19). As the severity of liver fibrosis escalates, the liver’s capacity to normally esterify fatty acids diminishes, leading to an increase in TG synthesis. Consequently, there is also a decline in the synthesis of phosphatidylcholine, an enzyme crucial for cholesterol esterification. This reduction in phosphatidylcholine synthesis results in decreased levels of total TC and TGs in the plasma. Additionally, in instances of liver malfunction, the synthesis of hydroxymethylglutaryl-CoA reductase, an enzyme integral to the synthesis of TC and TGs, also decreases, further exacerbating the reduction in TC and TG levels (19). Our findings revealed negative correlations between serum triglycerides/total cholesterol and liver parenchymal echogenicity score, indicating elevated lipid levels even in mild liver damage—a pattern consistent with pathological observations (20). We speculated that as WD progresses and liver fibrosis advances, the likelihood of observing increased TC and TG levels diminishes.

Notably, serum triglycerides and total cholesterol displayed negative correlations with spleen area score. Splenomegaly, indicated by an enlarged spleen area, often signifies various etiologies, predominantly infectious or non-infectious. Recent research (21) underscores the spleen’s role as a lipid metabolism reservoir, influencing lipid regulation through certain bioactive substances. Schmidt et al. (22) have likened the spleen to a reservoir for storing lipids. In instances of hypersplenism, splenic macrophages accumulate substantial quantities of fat due to their excessively active phagocytosis, ultimately resulting in hypolipidemia. An alternative explanation for the reduction in lipids could be the autoimmune activity of the mononuclear phagocytic system, which targets structures within HDL-cholesterol and LDL-cholesterol lipoproteins, leading to their removal from the plasma (23, 24). Furthermore, the “splenic factor” suggested by Asai et al. (25) may play a crucial role in regulating lipids. During splenomegaly, the overloaded splenic tissue enhances the impact of the splenic factor through phagocytosis, causing a significant increase in lipid storage and, consequently, a decrease in blood lipid levels (26).Thus, in WD patients with splenomegaly, lipid increases may be alleviated to some extent, supported by our study’s analysis.

#### 4.2.2 Liver enzymes

Significant disparities were observed in AST/ALT indicators between the groups with normal and abnormal blood lipids. Correlation analysis revealed a moderate negative correlation between serum triglycerides and AST/ALT, alongside a low degree of negative correlation between serum total cholesterol levels and AST/ALT. Hepatolenticular degeneration (WD) manifests cellular damage due to excessive copper and substance deposition (27). Even mild liver damage induces pathological shifts, such as increased liver cell membrane permeability, affecting cytoplasmic substance levels (28). ALT, predominantly cytoplasmic, leaks into the bloodstream, elevating blood ALT levels, while AST, mainly mitochondrial, decreases in mild liver damage due to unaffected mitochondria, resulting in a decreased AST/ALT ratio.

This study suggests that WD patients with a low AST/ALT ratio may exhibit a propensity for abnormal lipid metabolism. Mild to moderate liver damage, characterized by less mitochondrial involvement, correlates with elevated blood triglycerides and total cholesterol. Mild injury primarily impacts lipid breakdown, leading to increased blood lipids, while moderate injury affects lipid synthesis, reducing blood lipid levels.

#### 4.2.3 Blood glucose

Glucose serves as the primary energy source and precursor for various carbon-containing molecules in the body. It undergoes transformations into non-sugar substances, such as lipids, and can be converted back into glucose. Excess sugar is either stored as glycogen or undergoes sugar metabolism, catalyzing acetyl CoA into malonyl CoA, which synthesizes fatty acids and fat storage. However, in this study, no significant differences were found in blood glucose levels between the groups with normal and abnormal blood lipids, suggesting that dyslipidemia occurrence in WD patients may not be closely associated with glucose metabolism involvement.

#### 4.2.4 Nazer score

The Nazer score (4) evaluates significant liver function damage, indicative of severe liver damage, often warranting liver transplantation when the score ranges between 7 and 12. However, no significant differences were noted between the groups with normal and abnormal blood lipids in this study. Both groups exhibited Nazer scores below 1 point, suggesting that enrolled WD patients did not suffer liver function damage severe enough to warrant the Nazer score’s criteria. Hence, no significant intergroup differences were observed.

#### 4.2.5 Blood trace elements

Previous research has highlighted metabolic abnormalities in trace elements among individuals with hyperlipidemia (29). Despite hepatolenticular degeneration being associated with copper overload, the total serum copper content, including unbound copper, often decreases, mirroring ceruloplasmin levels. This observation prompts speculation that alterations in blood trace element levels might contribute to abnormal lipid metabolism in WD patients. However, in this study, no significant differences in blood trace elements were observed between the groups with normal and abnormal blood lipids. This outcome may be attributed to the study’s small sample size or the counteracting effects of various metal elements on blood lipids, which were not evident in the clinical blood lipid test results. Consequently, no notable distinctions emerged between the groups. Therefore, based on these findings, it is inferred that there is no significant correlation between abnormal lipid metabolism occurrence in WD patients and blood trace element levels.

## 5 Conclusion

The prevalence of abnormal blood lipid metabolism in hepatolenticular degeneration patients appears markedly higher than in the general population. Future extensive investigations can corroborate this finding. For individuals with unexplained dyslipidemia, particularly adolescents, screening for hepatolenticular degeneration is advisable.

Patients with mild to moderate liver injury and Wilson’s disease, characterized by limited mitochondrial involvement in liver cells, exhibit a propensity for elevated blood triglycerides and total cholesterol. Mild liver damage predominantly impacts lipid breakdown, resulting in increased blood lipids. Conversely, severe liver injury hampers lipid synthesis, leading to decreased blood lipid levels.

Dyslipidemia occurrence in hepatolenticular degeneration patients appears unrelated to glucose metabolism involvement.

No significant correlation was observed between abnormal lipid metabolism and blood trace element content in hepatolenticular degeneration patients.

## 6 Limitations

### 6.1 Incomplete research factors included

This retrospective study might have overlooked various factors affecting blood lipids due to its retrospective nature. Future comprehensive analyzes should incorporate a broader array of influencing factors through large-scale prospective studies.

### 6.2 Small sample size

The study’s relatively small sample size of 88 patients may have increased the likelihood of unidentified confounding factors, potentially skewing the results. Future longitudinal follow-up studies with larger sample sizes are warranted for more reliable outcomes.

### 6.3 Insufficient discriminatory power of liver function scoring table

The Nazer score, employed to assess liver function damage, proved inadequate in capturing the severity of liver function impairment in enrolled Wilson’s disease patients. Developing a more refined liver function scoring standard tailored to Chinese Wilson’s disease patients is suggested.

The absence of additional zoological and cellular experiments hinders the investigation and demonstration of the underlying causes of dyslipidemia in WD patients.

## Data Availability

The raw data supporting the conclusions of this article will be made available by the authors, without undue reservation.
